# A Method of Detections’ Fusion for GNSS Anti-Spoofing

**DOI:** 10.3390/s16122187

**Published:** 2016-12-19

**Authors:** Huiqi Tao, Hong Li, Mingquan Lu

**Affiliations:** Department of Electronic Engineering, Tsinghua University, Beijing 100084, China; thq12@mails.tsinghua.edu.cn (H.T.); lumq@mail.tsinghua.edu.cn (M.L.)

**Keywords:** GNSS, spoofing, detection, fusion, Dempster-Shafer theory, belief function

## Abstract

The spoofing attack is one of the security threats of systems depending on the Global Navigation Satellite System (GNSS). There have been many GNSS spoofing detection methods, and each of them focuses on a characteristic of the GNSS signal or a measurement that the receiver has obtained. The method based on a single detector is insufficient against spoofing attacks in some scenarios. How to fuse multiple detections together is a problem that concerns the performance of GNSS anti-spoofing. Scholars have put forward a model to fuse different detection results based on the Dempster-Shafer theory (DST) of evidence combination. However, there are some problems in the application. The main challenge is the valuation of the belief function, which is a key issue in DST. This paper proposes a practical method of detections’ fusion based on an approach to assign the belief function for spoofing detections. The frame of discernment is simplified, and the hard decision of hypothesis testing is replaced by the soft decision; then, the belief functions for some detections can be evaluated. The method is discussed in detail, and a performance evaluation is provided, as well. Detections’ fusion reduces false alarms of detection and makes the result more reliable. Experimental results based on public test datasets demonstrate the performance of the proposed method.

## 1. Introduction

The security of the Global Navigation Satellite System (GNSS) is deeply concerned with the rapid development of GNSS applications. Due to the weakness of the power and the openness of the signal characteristics, GNSS receivers are vulnerable to interferences, and the signals are easy to counterfeit. There have been several reports about successful GNSS spoofing, which could cause great damage to important infrastructures, such as the telecommunication network, the power grid, etc. Therefore, anti-spoofing technology attracts researchers’ attention [1].

There have been many methods of GNSS spoofing detection and exclusion [1,2,3,4]. They can be classified into three categories. The first can be named signal design. The typical method is cryptographic authentication. Encrypted military signals, such as the GPS P(Y) code and M code, could not be duplicated by the spoofer; however, their target user group is limited, and most commercial users do not have the authority to access these signals. Besides, cryptographic authentication is powerless to prevent repeat spoofing, which transmits the delayed copy of authentic signals. The second category can be called external aids. GNSS measurements and solutions can be checked with external systems, such as inertial measurement units (IMU), compass, cellular network positioning, etc. [3,5]. The external systems are independent of GNSS, and they are not affected by GNSS spoofing. However, external aids increase the complexity of the user receiver, and they are expensive to implement in general receivers. The third category can be called GNSS signal processing. There are many methods in this category, such as multi-antenna processing [6,7], signal power monitoring [8], correlation domain monitoring [9,10], vestigial signal detection, receiver autonomous integrity monitoring (RAIM) [4] and the extended method [11], etc. In addition, some crossing methods are proposed; for example, the authors of [12] propose a method based on machine learning and signal processing to monitor the interference of GNSS. However, there are shortcomings in these methods, and some of them are complementary in anti-spoofing. The cooperation of multiple spoofing detections is necessary to improve the performance of GNSS anti-spoofing.

Information fusion is a key issue of the cooperation of multiple spoofing detections. There are many methods to fuse the information of different detectors, such as Bayesian data fusion, the Kalman filtering method, Dempster–Shafer theory (DST), etc. However, there are some obstacles in the applications. For example, a priori knowledge of the detectors is needed for Bayesian data fusion. The statistical properties of most detectors are known in the case of authentic GNSS signals, but uncertain in the case of spoofing signals, i.e., a priori information of the GNSS spoofing detectors is partially unknown. The uncertainty makes it impossible to apply Bayesian inference to calculate the posterior probabilities of the possible results. For the Kalman filtering method, the precise estimate of the noise covariances, including the covariances of process noise and observation noise, is very important in applications. However, a precise estimate is hard to obtain in the case of spoofing, because the style of spoofing is varied and unknown. DST [13] is a general framework for reasoning with uncertainty, and it provides a solution to combine evidence from different sources. It is suitable for use in situations where a priori information of detectors is partially unknown. The authors of [14] propose the framework of a mathematic model for conjunct GNSS spoofing detection. The model is based on the DST. However, it does not give the way to valuate the basic probability assignment (BPA) or belief function for each possible result of detection. Thus, the method cannot be implemented in practice. This paper focuses on this problem of DST and proposes a method to overcome this difficulty.

Each spoofing detection makes a decision according to the anomaly of the signal. Usually, it is a hard decision to discern the authenticity of the received signals. A hard decision is easy to implement, but it loses some information of measurement. Thus, false decision cannot be rectified. Meanwhile, most spoofing detections are equivalent to hypothesis testings, and the test statistics are quantifications of the anomaly of measurements or signal features. The quantification of the anomaly is equivalent to a soft decision that indicates the degree of anomaly, and the soft decision can be used to evaluate the belief function in binary detection. This makes it possible to implement the DST if the frame of discernment is simplified properly while the belief function is constructed on the basis of the quantification of the anomaly.

This paper proposes a method to deal with this problem. There are two main points for the purpose of implementation. The first is to extract a simplified frame of discernment for spoofing detection. Compared with the existing method, some elements in the complete, but redundant frame of discernment are excluded, and only two basic and mutually exclusive elements are left. This is conducive to the valuation of belief functions. The second point and the main contribution of this work is to construct the belief function for each possible result of detections. The belief function is proposed based on the model of hypothesis testing for a spoofing detector. These two points make it feasible to fuse the information from multiple spoofing detections.

The remainder of this paper is organized as follows. Section 2 describes the method of spoofing detections’ fusion, including the model of detections’ fusion, the valuation of the belief function and performance of fusion. Section 3 provides a detailed performance evaluation of the proposed method. Simulations based on the fusion of two spoofing detections are given. Finally, the conclusions are provided in Section 4.

## 2. The Proposed Method of Spoofing Detections’ Fusion

A detailed discussion of the proposed method is provided in this section. First, a simplified frame of discernment is given. The simplification is for the purpose of the quantization for detection results. Then, the valuation of the belief function, which is the key of information fusion, is given and discussed. Finally, the performance of detections’ fusion is analyzed briefly.

### 2.1. The Model of Detections’ Fusion

Most of the spoofing detections focus on the anomalies of signal features or measurements. Generally, each detector aims at only one feature, and the result is evidence of the presence of the spoofing attack. However, this evidence would be inadequate and not absolute reliable in some cases. The combination of the results from multiple detectors is an approach to deal with this problem. A method based on DST to fuse multiple detectors is proposed in the following.

According to DST, a complete frame of discernment in spoofing detection should contain all possible mutually exclusive declared propositions, such as the noise, authentic signal, counterfeit signal and the mixture of them. They indicate the identity of the signal. However, this complete frame of discernment sets up obstacles for implementation. The main problem is the evaluation of BPA. Thus, it is necessary to simplify this frame for application. In what follows, the noise, authentic and counterfeit signal are denoted as “*N*”, “*A*” and “*C*”, respectively, and the frame of discernment is denoted as Θ.

In practice, noise is universal because the environment temperature cannot be absolute zero. The acquisition module of the GNSS receiver would determine the absence of satellite signals if there is only noise. Thus, *N* is redundant in Θ. In what follows, the object is limited to the satellite signal that has been processed by the receiver, regardless of its authenticity. In a broader sense, *C* does not only represent the spoofing signal, it also means the abnormal change of the satellite signal. Besides, *A* and *C* are exclusive for a signal. If authentic and counterfeit signals are mixed together in a processing channel and cannot be separated by the GNSS receiver, they should be marked as “abnormal”, and the identified result should be *C*. Thus, it is unnecessary to reserve a position for the mixture “AC” in Θ. Therefore, Θ could be simplified as follows:

(1) Θ={A,C}

That is, there are only two possible results of spoofing detector, “authentic” or “counterfeit”; the more generalized description is “no spoofing” or “spoofing”. This simplification of Θ facilitates the valuation of BPA and the belief functions, it also simplifies the combination rule. According to DST, for a possible result of one detector, the BPA is equivalent to the belief function because there are only two elements in Θ. Herein, the belief function is denoted as $m_{i}\left(\cdot \right)$, which corresponds to detector $D_{i}$. $m_{i}\left(\cdot \right)$ follows:

(2) $$\left\{\begin{array}{c} m_{i}\left(\phi \right)=0\\ m_{i}\left(A\right)+m_{i}\left(C\right)=1 \end{array}\right.$$

where *ϕ* represents an empty set. $m_{i}\left(\cdot \right)$ implies the believable degree of the evidence from detector $D_{i}$. For example, $D_{i}$ tends to determine the signal as “*A*” if $m_{i}\left(A\right)>m_{i}\left(C\right)$; otherwise, it tends to determine the signal as “*C*”. The conflict degree of different evidence can be defined as follows:

(3) $$\kappa =1-\left(\prod_{i=1}^{N}m_{i}\left(A\right)+\prod_{i=1}^{N}m_{i}\left(C\right)\right)$$

The combination result is reliable if all evidence is coincident, while it is unreliable when the conflict degree is high. κ=1 means that some evidence is completely opposite, such as $m_{1}\left(A\right)=1$ and $m_{2}\left(A\right)=0$.

Due to the inference of the combination rule of DST [13] in the case of binary detection, the combined m(·) are given as follows:

(4) $$\left\{\begin{array}{c} m\left(A\right)=\frac{\prod_{i=1}^{N}m_{i}\left(A\right)}{\;\;\prod_{i=1}^{N}m_{i}\left(A\right)+\prod_{i=1}^{N}m_{i}\left(C\right)\;\;}\\ m\left(C\right)=\frac{\prod_{i=1}^{N}m_{i}\left(C\right)}{\;\;\prod_{i=1}^{N}m_{i}\left(A\right)+\prod_{i=1}^{N}m_{i}\left(C\right)\;\;} \end{array}\right.$$

where *N* is the number of spoofing detectors. In the case that κ=1, there is $\prod m_{i}\left(A\right)+\prod m_{i}\left(C\right)=0$; m(·) cannot be obtained according to Equation (4). It is necessary to add a rule with Equation (4) in such a case as follows:

m(A)=m(C)=0.5whenκ=1

In what follows, they are denoted as Equation (4) together. After the fusion, a final decision must be made to determine the authenticity of the signal. The decision rule is given as follows:

(5) $$\left\{\begin{array}{c} \mathrm{Determine}\;\;A\;\;\mathrm{when}\;\;m(A)\geq m(C)\\ \mathrm{Determine}\;\;C\;\;\mathrm{when}\;\;m(A)<m(C) \end{array}\right.$$

As m(A)+m(C)=1, the rule of Equation (5) determines “*A*” if m(A)≥0.5 and determines “*C*” if m(A)<0.5. That is, 0.5 is the threshold of the final decision after detections’ fusion.

**Figure 1:**
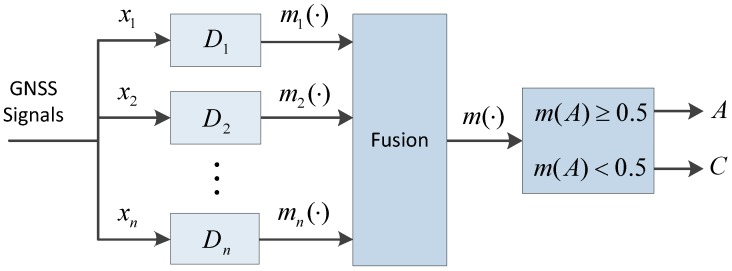
Diagram of the fusion of multiple spoofing detectors.

Figure 1 shows the diagram of the detections’ fusion. Detector $D_{i}$ points at $x_{i}$, which could be any feature of the GNSS signals or measurements, such as signal power, Doppler shifts, code delay, pseudoranges, the results of positioning, etc.

It should be noted that not the different detection methods, but the different signal features are the key of fusion. The evidence of the spoofing attack is collected from the detectors and fused according to the rule of Equation (4). The fusion makes the result more credible if the evidence from different detections is coincident, while it would give a compromise result according to all evidence if it is conflictive. Two examples are provided in Table 1 as follows to demonstrate the combination of coincident and conflictive evidence.

### 2.2. Valuation of the Belief Function for GNSS Spoofing Detections

Belief functions are the key to the detections’ fusion. They represent the reliability of the corresponding measurement or features of the signal. The belief function is calculated based on the basic probability assignment (BPA) in DST. As previously mentioned, BPA is equivalent to the belief function in the case of binary detections. However, there is no general way to get BPA or the belief function for a detection result. This section proposes a method to valuate the belief function and provides a brief discussion about two spoofing detections.

Most of the spoofing detections are equivalent to hypothesis testings. They can be described as:

(6) $$\left\{\begin{array}{cc} H_{0}: & T(x)\leq \gamma \\ H_{1}: & T(x)>\gamma \end{array}\right.$$

where $H_{0}$ and $H_{1}$ are hypotheses that represent the absence and presence of a spoofing attack, and they are equivalent to *A* and *C* of Equation (1), respectively. T(x) is the test statistic of *x*, which is a measurement of the signal. *γ* is the threshold of detection. Here, *x* could be any signal features and measurements, such as the absolute power or carrier-to-noise ratio ($C/N_{0}$) of the received signal, Doppler shift, outputs of the correlator, pseudorange measurement, etc.

The detection of Equation (6) makes a hard decision to decide whether the signal is authentic or not. It is easy to implement, but it loses some information and increases the error decision. T(x) is a quantization of the anomaly of *x*, and it also can be considered as a soft decision of the authenticity of signal. The belief function for the detection results of detector $D_{i}$ can be defined as:

(7) $$\left\{\begin{array}{c} m_{i}\left(A\right)=f\left(T_{i},\gamma_{i}\right)\\ m_{i}\left(C\right)=1-f\left(T_{i},\gamma_{i}\right) \end{array}\right.$$

where $m_{i}\left(\cdot \right)$ is the belief function, $T_{i}$ and $\gamma_{i}$ represent the test statistic and threshold of $D_{i}$, respectively, and *f* is the function that maps $T_{i}$ and $\gamma_{i}$ to a real number. The mapping is subject to:

(8) $$\left\{\begin{array}{c} f\left(T_{i},\gamma_{i}\right)\in \left[\;0,\;1\;\right]\\ m_{i}\left(A\right)>m_{i}\left(C\right)\;\;\mathrm{when}\;\;T_{i}<\gamma_{i}\\ m_{i}\left(A\right)<m_{i}\left(C\right)\;\;\mathrm{when}\;\;T_{i}>\gamma_{i}\\ m_{i}\left(A\right)=m_{i}\left(C\right)\;\;\mathrm{when}\;\;T_{i}=\gamma_{i} \end{array}\right.$$

This is in keeping with the decision rule of Equation (6). However, unlike Equation (6), $m_{i}\left(\cdot \right)$ of Equation (7) provides a soft decision of the authenticity of the signal. Compared with the hard decision, it retains more information of *x*. More than that, Equation (7) makes it possible to fuse the information from multiple detections to reduce the probability of error decisions.

There are many forms of *f* to meet Equation (8). The *p*-value of hypothesis testing would have more proper to reflect the reliability of the results than the test statistic and threshold. However, the calculation of the tail probability for each obtained statistics in the method of the *p*-value is too complicated. Herein, two definitions of *f* are proposed for simplicity in the application. In what follows, $T_{i}$ and $\gamma_{i}$ are assumed as non-negative, and this is possible in a proper way. One definition of *f* can be given as:

(9) $$f_{1}\left(T_{i},\gamma_{i}\right)={\left(\frac{1}{2}\right)}^{T_{i}/\gamma_{i}}$$

$f_{1}\left(T_{i},\gamma_{i}\right)$ is a decreasing function of $T_{i}/\gamma_{i}$, and it is easy to implement. However, $f_{1}\left(T_{i},\gamma_{i}\right)$ descends slowly even if the anomaly is obvious enough. Besides, $f_{1}\left(T_{i},\gamma_{i}\right)$ is in a half closed interval [0,1) because the base number of the power function is less than one. Another definition of *f* is given as:

(10) $$f_{2}\left(T_{i},\gamma_{i}\right)=\left\{\begin{array}{cc} 1-\frac{\;T_{i}\;}{\;2\gamma_{i}\;} & \;\;T_{i}<2\gamma_{i}\\ 0 & \;\;T_{i}\geq 2\gamma_{i} \end{array}\right.$$

**Figure 2:**
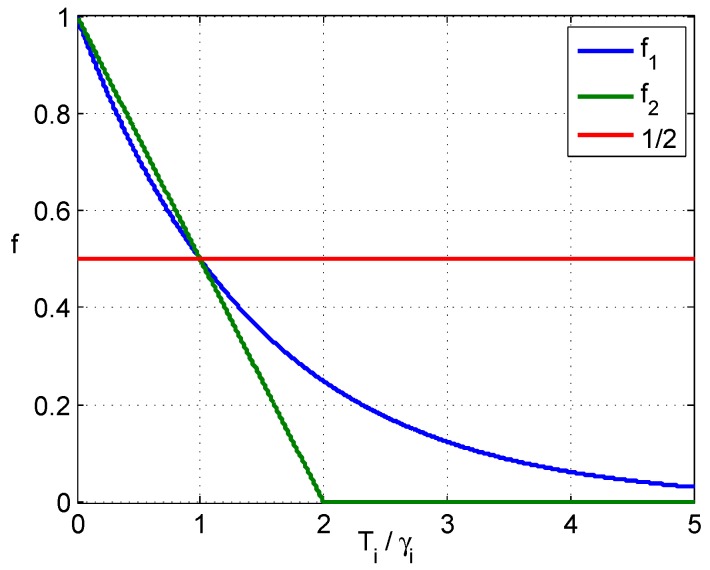
Curves of two $f(T_{i},\gamma_{i})$.

$f_{2}\left(T_{i},\gamma_{i}\right)$ meets Equation (8), as well, and it is in a closed interval [0,1]. However, $f_{2}\left(T_{i},\gamma_{i}\right)$ descends quickly, and it makes a decision $m_{i}\left(A\right)=0$ even if the anomaly is not obvious enough. The decision $m_{i}\left(A\right)=0$ is strong, and it makes an irrevocable decision while all other detectors are meaningless. Figure 2 shows the curves of $f_{1}\left(T_{i},\gamma_{i}\right)$ and $f_{2}\left(T_{i},\gamma_{i}\right)$, where $T_{i}/\gamma_{i}$ is plotted on the horizontal axis, and the red line is the threshold to determine “*A*” or “*C*”.

As mentioned previously, both $f_{1}\left(T_{i},\gamma_{i}\right)$ and $f_{2}\left(T_{i},\gamma_{i}\right)$ have weakness in practice. The linear combination of them would be applicable, and it can be given as follows:

(11) $$f\left(T_{i},\gamma_{i}\right)=\alpha \cdot f_{1}\left(T_{i},\gamma_{i}\right)+\left(1-\alpha \right)\cdot f_{2}\left(T_{i},\gamma_{i}\right)\;\;\;\;\;\;\;\;\alpha \in \left[\;0,\;1\;\right]$$

where *α* is the weight of $f_{1}\left(T_{i},\gamma_{i}\right)$; it can be set according to the actual implementation.

### 2.3. Performance of Detections’ Fusion

A spoofing detection can be fused with others by the method as mentioned previously, as long as it could be depicted by a hypothesis testing as Equation (6). There are two types of errors in hypothesis testings. One is false alarm and the other is missed detection. The former is the incorrect rejection of the true hypothesis $H_{0}$, while the latter is the failure to reject the false hypothesis $H_{1}$. The probabilities of these two errors can be described as follows:

(12) $$\left\{\begin{array}{c} P_{fa}=P\left(H_{1}|H_{0}\right)\\ P_{md}=P\left(H_{0}|H_{1}\right) \end{array}\right.$$

where $P_{fa}$ is the false alarm probability and $P_{md}$ is the missed detection probability. They are contradictory. For a given sample set, $P_{md}$ increases with the reduction of $P_{fa}$, and vice versa. $P_{fa}$ and $P_{md}$ cannot be reduced simultaneously unless the sample size increases, but this is infeasible especially in practice. Detection aiming at data set for a long time cannot reflect the real-time changes of signals. This limits the performance of single spoofing detection. However, it is different in case that multiple detections are fused, because the false alarms would not happen simultaneously in different detectors. This means that false alarms of fused detection would be much less than those of each detector.

**Figure 3:**
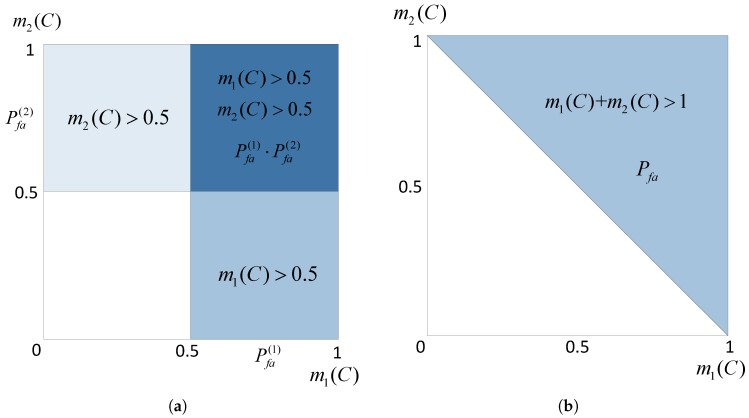
Regions of the false alarm in the case that detections are fused or not fused under hypothesis $H_{0}$. (**a**) Two detections are not fused; (**b**) two detections are fused.

Herein, a brief derivation of $P_{fa}$ is given in the case that only two detections are fused. The probabilities of $m_{i}\left(\cdot \right)$ under hypotheses $H_{0}$ and $H_{1}$ are denoted as $P_{0}^{\left(i\right)}$ and $P_{1}^{\left(i\right)}$, respectively. The false alarm probability of detector $D_{i}$ is denoted as $P_{fa}^{\left(i\right)}$, and the corresponding missed detection probability is denoted as $P_{md}^{\left(i\right)}$. In the case that only two detections are fused, m(C)>0.5 is equivalent to $m_{1}\left(C\right)+m_{2}\left(C\right)>1$, and m(A)≥0.5 is equivalent to $m_{1}\left(A\right)+m_{2}\left(A\right)\geq 1$. The derivation is provided in Appendix A. Based on Equations (4) and (5), $P_{fa}$ of fused detections can be given as follows:

(13) $$\begin{array}{ccc} P_{fa} & = & P\left(m\left(A\right)<0.5|H_{0}\right)\;=\;P\left(m\left(C\right)>0.5|H_{0}\right)\\ & = & P_{0}^{\left(1\right)}\left(m_{1}\left(C\right)>0.5\right)\cdot P_{0}^{\left(2\right)}\left(m_{2}\left(C\right)>1-m_{1}\left(C\right)|m_{1}\left(C\right)>0.5\right)\\ & & +\;P_{0}^{\left(2\right)}\left(m_{2}\left(C\right)>0.5\right)\cdot P_{0}^{\left(1\right)}\left(m_{1}\left(C\right)>1-m_{2}\left(C\right)|m_{2}\left(C\right)>0.5\right)\\ & & -\;P_{0}^{\left(1\right)}\left(m_{1}\left(C\right)>0.5\right)\cdot P_{0}^{\left(2\right)}\left(m_{2}\left(C\right)>0.5\right)\\ & < & P_{fa}^{\left(1\right)}+P_{fa}^{\left(2\right)}-P_{fa}^{\left(1\right)}\cdot P_{fa}^{\left(2\right)} \end{array}$$

where 0.5 is the threshold of m(·) to determine the authenticity of the signal. $P_{fa}^{\left(1\right)}+P_{fa}^{\left(2\right)}-P_{fa}^{\left(1\right)}\cdot P_{fa}^{\left(2\right)}$ is the false alarm probability of two detectors if their results are not fused together. In such a case, each false alarm would be mistaken for a spoofing attack no matter in which detector it happens. This can be explained as Figure 3.

The shadow areas in Figure 3a represent the region of false alarms under the hypothesis of $H_{0}$ in the case that two detections are not fused, while in Figure 3b, the shaded region is lessened in the case that two detectors are fused together. This is the graphical interpretation of the inequality (13). Meanwhile, the dark shaded area in Figure 3a, denoted as $R_{1}$, represents the region in which false alarms happen simultaneously in both $D_{1}$ and $D_{2}$. Denoting the shaded area in Figure 3b as $R_{2}$, $R_{1}$ is a subset of $R_{2}$, i.e., $R_{1}\subset R_{2}$. Thus, there is:

(14) $$P_{fa}^{\left(1\right)}\cdot P_{fa}^{\left(2\right)}<P_{fa}$$

The above inequality can be deduced as follows:

(15) $$\begin{array}{ccc} P_{fa} & = & P\left(m_{1}\left(C\right)+m_{2}\left(C\right)>1|H_{0}\right)\\ & = & P_{0}^{\left(1\right)}\left(m_{1}\left(C\right)<0.5\right)\cdot P_{0}^{\left(2\right)}\left(m_{2}\left(C\right)>1-m_{1}\left(C\right)|m_{1}\left(C\right)<0.5\right)\\ & & +\;P_{0}^{\left(2\right)}\left(m_{2}\left(C\right)<0.5\right)\cdot P_{0}^{\left(1\right)}\left(m_{1}\left(C\right)>1-m_{2}\left(C\right)|m_{2}\left(C\right)<0.5\right)\\ & & +\;P_{0}^{\left(1\right)}\left(m_{1}\left(C\right)>0.5\right)\cdot P_{0}^{\left(2\right)}\left(m_{2}\left(C\right)>0.5\right)\\ & & >\;P_{fa}^{\left(1\right)}\cdot P_{fa}^{\left(2\right)} \end{array}$$

Based on Equations (13) and (14), the range of $P_{fa}$ can be given as:

(16) $$P_{fa}^{\left(1\right)}\cdot P_{fa}^{\left(2\right)}<P_{fa}<P_{fa}^{\left(1\right)}+P_{fa}^{\left(2\right)}-P_{fa}^{\left(1\right)}\cdot P_{fa}^{\left(2\right)}$$

Meanwhile, $P_{md}$ of fused detections can be given as follows:

(17) $$\begin{array}{ccc} P_{md} & = & P\left(m\left(A\right)\geq 0.5|H_{1}\right)\\ & = & P_{1}^{\left(1\right)}\left(m_{1}\left(A\right)\geq 0.5\right)\cdot P_{1}^{\left(2\right)}\left(m_{2}\left(A\right)\geq 1-m_{1}\left(A\right)|m_{1}\left(A\right)\geq 0.5\right)\\ & & +\;P_{1}^{\left(2\right)}\left(m_{2}\left(A\right)\geq 0.5\right)\cdot P_{1}^{\left(1\right)}\left(m_{1}\left(A\right)\geq 1-m_{2}\left(A\right)|m_{2}\left(A\right)\geq 0.5\right)\\ & & -\;P_{1}^{\left(1\right)}\left(m_{1}\left(A\right)\geq 0.5\right)\cdot P_{1}^{\left(2\right)}\left(m_{2}\left(A\right)\geq 0.5\right)\\ & < & P_{md}^{\left(1\right)}+P_{md}^{\left(2\right)}-P_{md}^{\left(1\right)}\cdot P_{md}^{\left(2\right)} \end{array}$$

where $P_{md}^{\left(1\right)}+P_{md}^{\left(2\right)}-P_{md}^{\left(1\right)}\cdot P_{md}^{\left(2\right)}$ is the missed detection probability of two detectors if their results are not fused together. Each missed detection cannot be re-examined in the case that two detectors are not fused. Similar to Equation (14), there is:

(18) $$P_{md}>P_{md}^{\left(1\right)}\cdot P_{md}^{\left(2\right)}$$

Based on Equations (17) and (18), the range of $P_{md}$ can be given as:

(19) $$P_{md}^{\left(1\right)}\cdot P_{md}^{\left(2\right)}<P_{md}<P_{md}^{\left(1\right)}+P_{md}^{\left(2\right)}-P_{md}^{\left(1\right)}\cdot P_{md}^{\left(2\right)}$$

The range of $P_{fa}$ and $P_{md}$, Equations (16) and (19), just provides rough boundaries of $P_{fa}$ and $P_{md}$. They clearly show that both $P_{fa}$ and $P_{md}$ in the case of fusion are less than the corresponding values in the case that detections are not fused. The actual ranges depend on the performance of the detectors, which are fused. Monte Carlo simulations based on two detectors are given in the following section to demonstrate the performance of fusion.

The above discussions are based on the fusion of two detectors. Actually, the combination rule of Equation (4) is associative, that is:

(20) $$m_{1}⊕m_{2}⊕m_{3}=m_{1}⊕\left(m_{2}⊕m_{3}\right)=m_{1}⊕m_{2}^{\prime }$$

where ⊕ represents the operation of the combination and $m_{2}^{\prime }$ represents the combination of $m_{2}$ and $m_{3}$. The derivation of the associative law is provided in Appendix B. Equation (20) means that the combination of multiple detections can be equivalent to the combination of two detections after some of them are fused together. Thus, discussions about two detections are sufficient to extend to the cases of multiple detections.

## 3. Performance Evaluation

A model of detections’ fusion has been given, and the valuation of the belief function has been proposed in the above section. In order to evaluate the performance of the method, simulations based on the fusion of two spoofing detections are provided in this section.

### 3.1. Two Detectors

Herein, two detectors, monitoring the correlator outputs and consistency of Doppler, are given. They aim at different features of GNSS signal and provide different evidence of the authenticity of the signal.

#### 3.1.1. Monitoring of Correlator Outputs

Monitoring of the received signal power is a usual method of spoofing detection, and it can be implemented in several ways, such as the monitoring of absolute power, $C/N_{0}$ and the correlator outputs [8]. The anomaly of correlator outputs reflects the abnormal change of the power of received GNSS signals. Some methods have been proposed to monitor the correlator outputs, such as testing the goodness of fit based on the $\chi^{2}$ distribution [10,15], Herein, another practical way of correlator output monitoring is proposed.

In the case of the spoofing attack, it is difficult for the spoofer to calibrate the power of the spoofing signal to maintain a stable level at the target receiver because of the uncertainty of the relative motion between spoofer and receiver. Besides, correlator outputs may be abnormal because the tracking loop is disturbed by spoofing. Monitoring the stationarity of correlation values is a practical way to detect the anomaly of the signal. Denote the correlator outputs in time *k* as r[k]. There are jumps in correlator outputs because of the change of navigation data. Denote x[k] as:

(21) x[k]=D[k]·r[k]

where D[k] represent the navigation data and its value is one or −1. Then, x[k] can be approximated as a Gaussian random variable because it is determined by several factors, such as signal power, tracking errors, noise, etc. Assuming that the signal is locked and the parameters of the receiver do not change, the mean value of x[k] reflects the power of the signal while the variance of x[k] represents other effects, such as noise and errors. The outputs of the correlator are steady in the case of authentic signals and unsteady in the case of the spoofing attack. Denote two sample sets of correlator outputs in different times as X={x[1],x[2],⋯,x[K]} and Y={y[1],y[2],⋯,y[K]}; they conform to a Gaussian distribution as:

(22) $$\left\{\begin{array}{c} X\;∼\;N(\mu_{x},\;\sigma_{x}^{2})\\ Y\;∼\;N(\mu_{y},\;\sigma_{y}^{2}) \end{array}\right.$$

As mentioned previously, the mean value reflects the power of the signal, while the variance represents the effects of random factors. Therefore, the spoofing detection based on the monitoring of correlator outputs can be equivalent to the following hypothesis testing:

(23) $$\left\{\begin{array}{cc} H_{0}: & \mu_{x}=\mu_{y}\\ H_{1}: & \mu_{x}\neq \mu_{y} \end{array}\right.$$

Assuming $\sigma_{x}^{2}=\sigma_{y}^{2}=\sigma^{2}$ and because $\sigma^{2}$ is unknown, define the test statistic as follows:

(24) $$T=\sqrt{K}\cdot \frac{\bar{x}-\bar{y}}{\sqrt{{\hat{\sigma }}_{x}^{2}+{\hat{\sigma }}_{y}^{2}\;}\;\;}$$

where $\bar{x}$ and $\bar{y}$ are the mean value of *X* and *Y* with a data length of *K* and ${\hat{\sigma }}_{x}^{2}$ and ${\hat{\sigma }}_{y}^{2}$ are estimations of *X* and *Y*, respectively. $({\hat{\sigma }}_{x}^{2}+{\hat{\sigma }}_{y}^{2})/2$ is an unbiased estimation of the variance of sample group {X,Y}; $\bar{x}$ and ${\hat{\sigma }}_{x}^{2}$ are given as:

(25) $$\left\{\begin{array}{c} \bar{x}=\frac{1}{K}\sum_{k=0}^{K-1}x\left[k\right]\\ {\hat{\sigma }}_{x}^{2}=\frac{1}{K}\sum_{k=0}^{K-1}{\left(x\left[k\right]-\bar{x}\right)}^{2} \end{array}\right.$$

$\bar{y}$ and ${\hat{\sigma }}_{y}^{2}$ can be obtained in the same way. The test statistic as Equation (24) has been used for GPS interference detection [16]. Under hypothesis $H_{0}$, the test statistic conforms to Student’s *t* distribution with 2K−2 degrees of freedom [17] as follows:

(26) T∼t(2K−2)

Thus, Equation (23) is equivalent to:

(27) $$\left\{\begin{array}{cc} H_{0}: & |T|\leq \gamma \\ H_{1}: & |T|>\gamma \end{array}\right.$$

where *γ* is the threshold of detection, and it can be given as follows:

(28) $$\gamma =Q_{t(2K-2)}^{-1}\left(1-P_{fa}/2\right)$$

where $Q_{t(2K-2)}^{-1}\left(\cdot \right)$ represents the inverse function of the cumulative density function (CDF) of Student’s *t* distribution.

#### 3.1.2. Consistency Check of Doppler

Doppler shift is generated by the relative motion of the satellite and receiver. The carrier Doppler shift of the GNSS signal must be consistent with its code Doppler shift. However, this is not satisfied in some cases of the spoofing attack. Thus, the consistency check of Doppler can be used for spoofing detection. A brief introduction of this method is given herein.

As a consequence of the formula of Doppler shift, the pseudo-code Doppler of the GNSS signal is proportional to the carrier Doppler; the proportionality coefficient is given as follows:

(29) $$\alpha =\frac{f_{D\_code}}{f_{D\_carrier}}=\frac{f_{code}}{f_{carrier}}$$

where $f_{D\_code}$ is the Doppler shift of pseudo-code and $f_{D\_carrier}$ is the Doppler shift of the carrier and $f_{D\_code}$. $f_{code}$ is the code rate of the pseudo-code, and $f_{carrier}$ is the carrier frequency. For example, the coefficient of the GPS L1 C/A signal is:

(30) $$\alpha =\frac{f_{D\_code}}{f_{D\_carrier}}=\frac{1.023\times 10^{6}}{1575.42\times 10^{6}}=\frac{1}{1540}$$

Not just the Doppler of the pseudo-code and carrier, but a similar relationship exists between signals of different frequencies from one satellite, so the consistency check can apply to them. Based on Equation (29), define:

(31) $$x=f_{D\_code}-\alpha f_{D\_carrier}$$

The equivalent hypothesis testing of the consistency check of Doppler can be written as follows:

(32) $$\left\{\begin{array}{cc} H_{0}: & x[k]=w[k]\\ H_{1}: & x[k]=A+w[k] \end{array}\right.$$

*A* is a non-zero bias between carrier Doppler and code Doppler, and w[k] represents the noise and error of measurements. Assuming that w[k] conforms to a zero mean Gaussian distribution, x[k] can be described as:

(33) $$x\;∼\;N(\mu ,\;\sigma^{2})$$

where *μ* and $\sigma^{2}$ represent the mean value and variance. Hypothesis testing of Equation (32) is equivalent to:

(34) $$\left\{\begin{array}{cc} H_{0}: & \mu =0\\ H_{1}: & \mu \neq 0 \end{array}\right.$$

As $\sigma^{2}$ is unknown, define the test statistic as follows:

(35) $$T=\frac{\;\;\sqrt{K}\cdot \bar{x}\;\;}{\hat{\sigma }}$$

where $\bar{x}$ is the mean value of the observed data of x[k] with data length *K*, and $\hat{\sigma }$ represents the maximum likelihood estimation (MLE) of *σ*; they can be obtained as Equation (25). The test statistic conforms to Student’s *t* distribution with K−1 degrees of freedom [17] under hypothesis $H_{0}$ as follows:

(36) T∼t(K−1)

Therefore, the detection as Equation (27) can be used for the consistency check of Doppler, as well as the threshold is obtained by a similar way as Equation (28).

### 3.2. Simulation Results

There are two parts of the simulations in this section. The first is the Monte Carlo simulation of the performance of fusion, and the second aims at public test datasets. Monitoring of correlator outputs and the consistency check of Doppler shifts, which have been discussed previously, are employed and fused together in what follows. Their test statistics are denoted as $T_{1}$ and $T_{2}$, which correspond to Equations (24) and (35). The corresponding belief functions are denoted as $m_{1}\left(\cdot \right)$ and $m_{2}\left(\cdot \right)$, respectively. Both detectors adopt the same false alarm probability denoted as $P_{fa}^{\left(i\right)}$.

#### 3.2.1. Probabilities of False Alarm and Detection

The performance of a detector can be depicted by the receiver operating characteristics (ROC) curve, which illustrates the relationship of false alarm probability $P_{fa}$ and detection probability $P_{d}$. Here, detection probability $P_{d}$ can be obtained as:

(37) $$P_{d}=P\left(H_{1}|H_{1}\right)=1-P\left(H_{0}|H_{1}\right)=1-P_{md}$$

**Figure 4:**
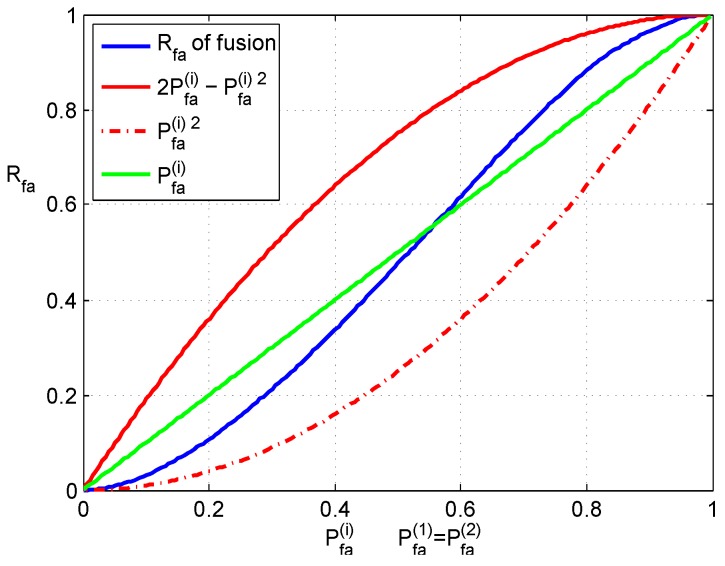
False alarm probability of fusion.

**Figure 5:**
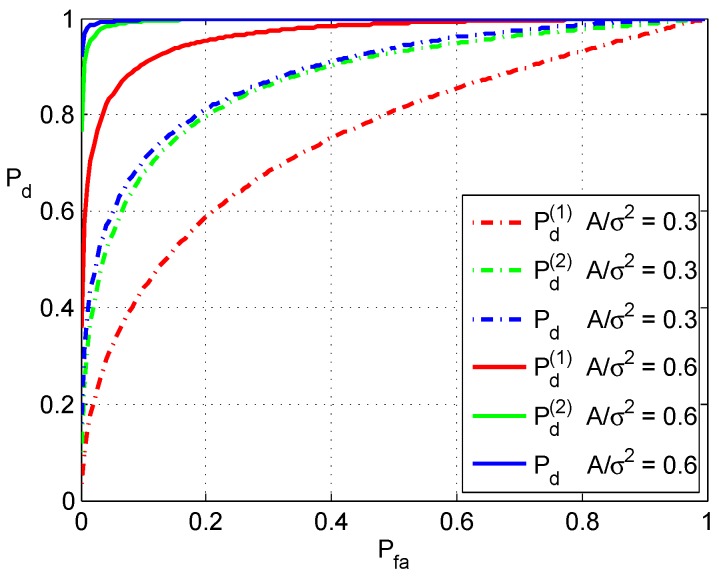
ROC curves of detections.

In the following Monte Carlo simulations, a dataset that conforms to a standard normal distribution is employed. The false alarm probability of fused detection is counted based on the detection results. Then, a bias, denoted as *A*, is added to the dataset to simulate the anomaly of measurements. For the first detector, *A* is added to the difference of mean value $\bar{x}-\bar{y}$. The results of two detectors and fused detection are counted. The results are shown in Figure 4 and Figure 5.

Figure 4 shows the simulation results of false alarms. The horizontal axis represents $P_{fa}^{\left(i\right)}$, while the vertical axis shows the rate of false alarms that happened in simulations. Here, the rate of false alarm is denoted as $R_{fa}$; it is tallied in Monte Carlo simulations. Two red curves in Figure 4 give the rough bounds of $P_{fa}$, which has been described in Equation (16). The blue curve is the $R_{fa}$ of fusion. It can be observed that fused $R_{fa}$ is between two boundaries. Moreover, in a practical range, $R_{fa}$ is less than $P_{fa}^{\left(i\right)}$, which is plotted in the green curve, and this means that false alarms are reduced by fusion.

ROC curves are plotted in Figure 5. In the simulations, two different normalized biases *A* are tested, and the corresponding results are plotted in dashed lines and solid lines. In both cases, blue curves are higher than the corresponding red and green curves. This means that the detection performance is improved by the fusion. It should be noted that the ROC curves of fusion take account of the reduction of false alarms, which is shown in Figure 4. Simulation results with other $A/\sigma^{2}$ are similar to them.

Actually, the improvement of detection performance is reasonable, because the data size of multiple detections’ fusion is much larger than the data size of single detection, while the fusion does not extend the time of detection.

#### 3.2.2. Test Results on TEXBAT

Simulations of multiple detections’ fusion based on the proposed method are provided next. The test data are from TEXBAT, which is a public dataset of eight high-fidelity digital recordings of live static and dynamic GPS L1 C/A spoofing tests. It is conducted by the Radio Navigation Laboratory of the University of Texas at Austin, and each scenario in TEXBAT has been described in detail in [18]. In Scenario 1 of TXBAT, the counterfeit signals substitute all authentic GNSS signals instantaneously; the power of counterfeit signals is much weaker than the authentic. In Scenario 2, the spoofer increases the power of the counterfeit signal, and it has a 10-dB power advantage; this is an obvious power attack. Moreover, the spoofer changes the counterfeit signals’ carrier phase proportionally when it shifts the code phase to induce a position or timing deviation in the target receiver. The power advantage of counterfeit signals is common in the next scenarios, but it is reduced. In Scenario 3, the spoofer fixes the carrier phase offset between the counterfeit signals and the authentic signals when it shifts the code phase of counterfeit signals. Scenario 4 is identical to Scenario 3, except that the power advantage of the counterfeit signal is reduced again. Static Scenarios 1∼4 are tested, and the results are discussed in this section. In each scenario, the detection results of just one satellite, GPS PRN 6, is discussed because the results of most satellites in the same scenario are similar.

In the following tests, a software-defined GPS receiver is employed. Common acquisition and tracking algorithms of GPS signals are implemented in the receiver where a phase-locked loop (PLL) is used for carrier tracking and a common delay lock loop (DLL) is used for code tracking. The coherent integration time is 1 ms in the receiver, and a measurement is obtained per one millisecond. The detection time is 50 milliseconds as the sample size is K = 50. The time interval of two sample sets, X and Y, is 1 s. This time interval can be set according to the application scenario. Herein, the short interval is conducive to improve the real-time capability of detectors. Besides, false alarm probabilities or significance levels are set as $P_{fa}=0.005$ in both detections.

**Figure 6:**
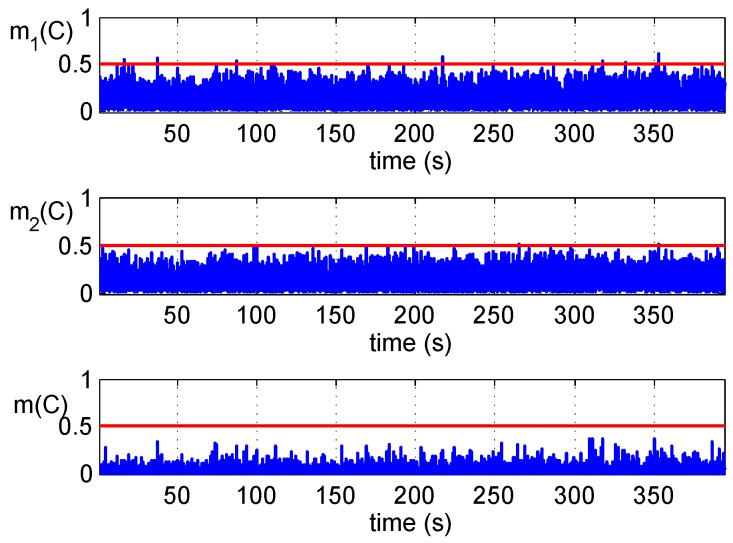
Detections’ fusion of the clean static dataset.

Figure 6 shows the test results of the clean static dataset where the GPS signals are not disturbed by counterfeit signals. It can be observed that there is a small number of false alarms in both two detections, and they disappear in the fused results. Reduction of false alarms is coincident with the curve in Figure 4, and it suggests that higher $P_{fa}^{\left(i\right)}$ can be adopted to obtain a lower $P_{md}^{\left(i\right)}$ in each detector before the fusion.

**Figure 7:**
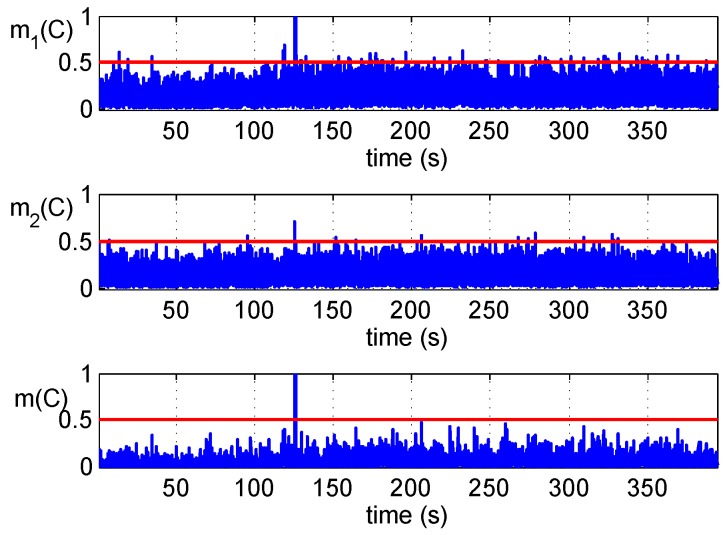
Detections’ fusion of the Scenario 1 dataset.

Test results of Scenario 1 for GPS PRN 6 are shown in Figure 7. The sudden replacement of the signals and the power difference disturbed the correlator and tracking loop. It can be observed that an obvious change of correlator outputs occurs at about 125 s in the time history, and an anomaly between carrier Doppler and code Doppler is detected at the same time, although the signal is tracked and locked by the receiver in the whole process.

**Figure 8:**
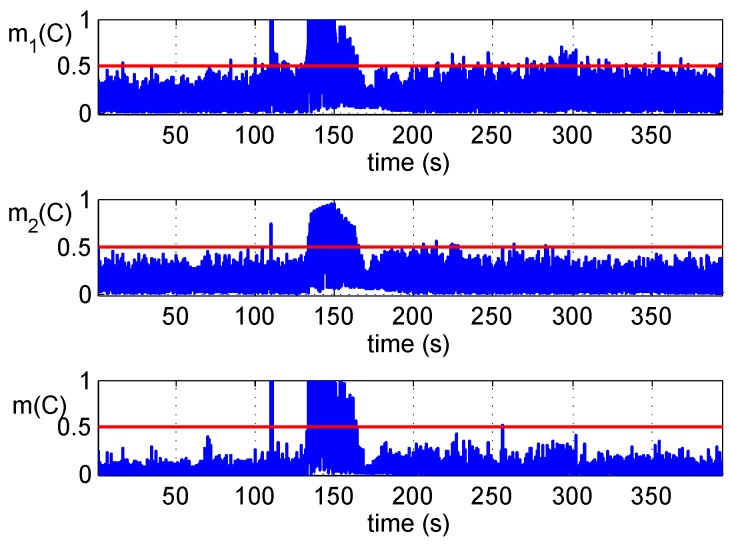
Detections fusion of the Scenario 2 dataset.

Figure 8 shows the fused results of two detections of Scenario 2. They are similar to the results of Scenario 1; the appearance of counterfeit signals with the power advantage makes two detectors issue alarms simultaneously at about 110 seconds. Moreover, with the shift of the carrier phase and code phase, the consistency of carrier Doppler and code Doppler is not met. Meanwhile, correlator outputs are not stationary because the tracking loop is disturbed. These are reflected in the figure.

**Figure 9:**
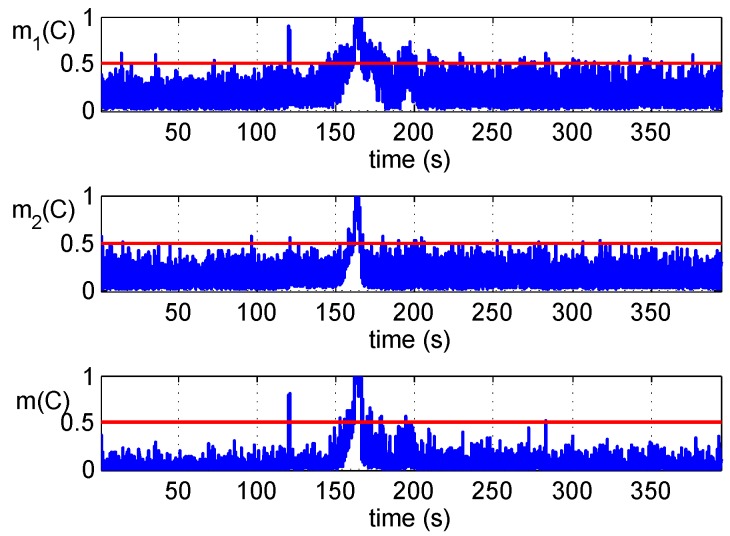
Detections’ fusion of the Scenario 3 dataset.

**Figure 10:**
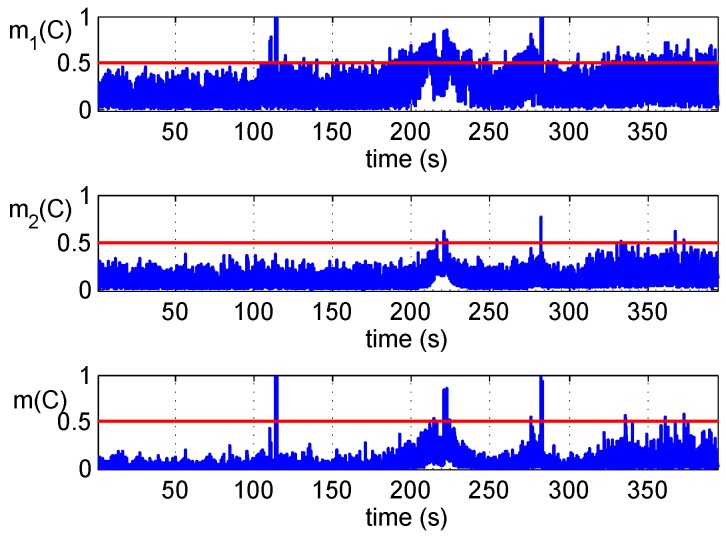
Detections’ fusion of the Scenario 4 dataset.

The fused results based on Scenarios 3 and 4 are shown in Figure 9 and Figure 10, respectively. In Scenario 3, the spoofer’s power advantage is weaker than the case of Scenario 2, but it still can be detected by the monitoring of correlator outputs. Besides, it can be observed in Figure 10 that the fused result exceeds the threshold at about 160 seconds, even though the second detector does not raise the alarm. This shows the robustness of the fusion method.

It can be observed from the above results that decisions after the fusion are more distinguishable than before the fusion. It should be noted that stable tracking conditions are crucial to obtain the test statistics; however, the stability of tracking cannot be guaranteed under the spoofing attack. Actually, in the above tests, tracking conditions are stable for most of the time, except sometimes in the tests of Scenarios 3 and 4. In test of Scenario 3, the receiver failed to track and lock the signal for about fifteen seconds at about 160 s in the time history. In this period, the receiver attempted to track and lock the signal; the test statistics based on the outputs of tracking loop cannot reflect the real conditions. However, failure to lock the signal indicates the anomalies of the signal. It is reasonable that detectors issue alarms to declare the unavailability of the signal in this case.

The above simulations illustrate the process and effect of the detections’ fusion. It can be observed on them that the fusion of multiple detections reduces the false alarms and makes the results more reliable. Only two detectors are fused in this section. However, a complete anti-spoofing method should employ more detectors and fuse them together.

## 4. Conclusions

As long as a spoofing detection could be depicted as a hypothesis testing, it can be fused with others by the method that is proposed in this paper. A key point of the fusion method is to simplify the discernment frame in order to assign the belief functions for spoofing detection results. Most of the spoofing detections are equivalent to hypothesis testings. The deviation degree of the test statistic and threshold represents the reliability of the measurement, and it is the basis of the belief function of the spoofing detection result. The performance evaluation and simulations based on two detections are provided in the paper. The proposed method improves the performance of spoofing detection and makes the results more reliable. The root cause of improvement is the increase of the detection data. However, there are some questions to be solved; for example, the evidence from different detections should be independent, but this may not be true in spoofing detections because some measurements are related in the GNSS receiver, such as $C/N_{0}$ and correlation values, while some measurements are independent, such as $C/N_{0}$ and Doppler shift. Besides, the selection of detectors to be fused in a specific scenario and the assignment of weightings for them are problems to be studied.

## Figures and Tables

**Table 1:** Examples for combination rule.

Coincident Evidence				Conflictive Evidence			
	$m_{1}$	$m_{2}$	$m_{1}⊕m_{2}$		$m_{1}$	$m_{2}$	$m_{1}⊕m_{2}$
A	0.8	0.2	0.903	A	0.8	0.2	0.632
C	0.2	0.3	0.097	C	0.3	0.7	0.368

## Appendix A

In the case that only two detections are fused, m(C)>0.5 is equivalent to $m_{1}\left(C\right)+m_{2}\left(C\right)>1$, and m(A)≥0.5 is equivalent to $m_{1}\left(A\right)+m_{2}\left(A\right)\geq 1$. The derivation is given as follows:

(A1) $$\begin{array}{c} m\left(C\right)=\frac{m_{1}\left(C\right)m_{2}\left(C\right)}{\;\;m_{1}\left(A\right)m_{2}\left(A\right)+m_{1}\left(C\right)m_{2}\left(C\right)\;\;}>0.5\\ ⇕\\ m_{1}\left(C\right)m_{2}\left(C\right)>m_{1}\left(A\right)m_{2}\left(A\right)\\ ⇕\\ m_{1}\left(C\right)m_{2}\left(C\right)>\left(1-m_{1}\left(C\right)\right)\left(1-m_{2}\left(C\right)\right)\\ ⇕\\ m_{1}\left(C\right)+m_{2}\left(C\right)>1 \end{array}$$

where “⇕” means “be equivalent to”. Similarly, there is:

(A2) $$\begin{array}{ccc} m\left(A\right)=\frac{m_{1}\left(A\right)m_{2}\left(A\right)}{\;\;m_{1}\left(A\right)m_{2}\left(A\right)+m_{1}\left(C\right)m_{2}\left(C\right)\;\;}\geq 0.5 & \Leftrightarrow & m_{1}\left(A\right)+m_{2}\left(A\right)\geq 1 \end{array}$$

## Appendix B

The combination rule of Equation (4) is associative. The derivation is given as follows:

(B1) $$\begin{array}{c} m_{1}\left(A\right)⊕\left(m_{2}\left(A\right)⊕m_{3}\left(A\right)\right)\\ \;\;\;\;=\frac{m_{1}\left(A\right)\frac{m_{2}\left(A\right)m_{3}\left(A\right)}{m_{2}\left(A\right)m_{3}\left(A\right)+m_{2}\left(C\right)m_{3}\left(C\right)}}{\;\;\;m_{1}\left(A\right)\frac{m_{2}\left(A\right)m_{3}\left(A\right)}{m_{2}\left(A\right)m_{3}\left(A\right)+m_{2}\left(C\right)m_{3}\left(C\right)}\;+\;m_{1}\left(C\right)\frac{m_{2}\left(C\right)m_{3}\left(C\right)}{m_{2}\left(A\right)m_{3}\left(A\right)+m_{2}\left(C\right)m_{3}\left(C\right)}\;\;\;}\\ \;\;\;\;=\frac{m_{1}\left(A\right)m_{2}\left(A\right)m_{3}\left(A\right)}{\;\;\;m_{1}\left(A\right)m_{2}\left(A\right)m_{3}\left(A\right)+m_{1}\left(C\right)m_{2}\left(C\right)m_{3}\left(C\right)\;\;\;}\\ \;\;\;\;=\;m_{1}\left(A\right)⊕m_{2}\left(A\right)⊕m_{3}\left(A\right) \end{array}$$

Similarly, there is:

(B2) $$m_{1}\left(C\right)⊕\left(m_{2}\left(C\right)⊕m_{3}\left(C\right)\right)=m_{1}\left(C\right)⊕m_{2}\left(C\right)⊕m_{3}\left(C\right)$$
